# A New Method for Assessing Patients’ Obesity-Associated Infection Risk Using X-rays in Hip Arthroplasties

**DOI:** 10.3390/jcm12237277

**Published:** 2023-11-24

**Authors:** Sebastian Breden, Florian Hinterwimmer, Simone Beischl, Sarah Consalvo, Alexandra S. Gersing, Ulrich Lenze, Rüdiger von Eisenhart-Rothe, Carolin Knebel

**Affiliations:** 1 Department of Orthopedics and Sports Orthopedics, Klinikum Rechts der Isar, Technical University of Munich, 81675 Munich, Germany; 2 Institute for AI and Informatics in Medicine, Technical University of Munich, 81675 Munich, Germany; 3 Department of Neuroradiology, University Hospital Munich, 81675 Munich, Germany

**Keywords:** THA, BMI, periprosthetic infection, adiposity

## Abstract

Overweight patients have higher complication rates during and after surgical procedures. In total hip arthroplasty (THA), postoperative infection is a major complication. In this study, we show that the patient’s body mass index (BMI) can be approximated by a newly developed grading system using preoperative X-rays. Furthermore, we show that a higher score and BMI result in a higher risk of infection. For this retrospective study, 635 patients undergoing THA or revision surgeries in 2018 and 2019 were included. The preoperatively acquired X-rays of the pelvis were analyzed using a four-stage grading system. The infection rate was compared to our score and the patients’ BMI. The mean BMI (95% confidence) of all patients graded as grade 0 was 25.16 (24.83; 25.50) kg/m^2^, for grade 1, it was 30.31 (29.52; 31.09) kg/m^2^, for grade 2, it was 35.06 (33.59; 36.54) kg/m^2^, and it was 45.03 (39.65; 50.41) kg/m^2^ for grade 3. The risk of infection was 4% in patients with normal radiographs, rising from 7% in patients graded as 1 up to 18% in each of the highest categories. This study shows that we were able to create a semi-quantitative grading tool for the abdominal contour displayed on X-rays of the pelvis in order to estimate the patients’ BMI and therefore the infection rate. A higher abdominal contour grade showed higher infection rates at follow-up.

## 1. Introduction

Obesity is a growing burden to our society, with an increase in the proportion of overweight persons worldwide of around 8% between 1980 and 2013 [1]. According to a recent study, in 2020, 53.5% of adults in Germany were overweight (BMI > 25 kg/m^2^) and 19.0% were obese (BMI > 30 kg/m^2^) [2]. This poses a major challenge for our health system. A higher weight not only affects the development of diseases, but also influences the outcome of surgical interventions, among others, by triggering infections [3].

Surgical site infections are a major complication in all cutting disciplines; in particular, infections of and around metal implants occur regularly and are difficult to treat [3]. The incidence of deep wound infection after primary total hip arthroplasty ranges around 1% [4], with an even higher quota in revision surgeries [5]. Whereas postsurgical infections can have different causes, it has been shown that overweight patients are at higher risk after every surgical intervention [3,6].

The etiology of higher rates of infections in overweight patients is still under discussion. A much favored approach explains this phenomenon with a deranged immune response [7]. Additionally, correlations to bad wound healing because of changes in the skin have been discussed [8].

Total hip replacement is one of the most performed surgeries of our time [9], with the number of interventions still growing [10]. A common mode of failure in hip arthroplasty is infection [11]. Whereas the rate of infections is diminishing, the financial burden of revision surgeries is rising [12]. One predictor of a higher rate of periprosthetic joint infection is obesity [13,14,15,16].

We want to develop an easily obtainable score to estimate the risk of perioperative infection in hip surgeries. Whilst the known body mass index (BMI) is the most common tool to assess a person’s weight status, there are voices stating that BMI is not the ideal tool to predict increased morbidity [17]. The sole calculation of BMI does not account for the amount of fat around the waist, which is why body circumference is often proclaimed as superior to BMI when assessing cardiac risk [17].

Another problem with estimating the higher perioperative risk of infection due to obesity using BMI is a lack of information or the patient’s tendency to underestimate their weight as a report bias [18]. 

To account for these factors and to give an easy and unbiased objectification of obesity and therefore the risk of infection, we tried to establish a new classification system, which uses only preoperative X-rays that have already been taken in preparation before every surgery to be performed on the hip.

We hypothesize that the amount of overhanging abdominal fat correlates with BMI and can be used to estimate the risk of postoperative infections in THA or hip prosthesis revisions.

The aim of this work is to establish a new score to assess patient obesity that can be used to estimate a patient’s risk of postoperative infection after surgeries concerning hip prostheses.

This study is split into two parts: first, we wanted to validate our new method by comparing it to BMI measurements, and secondly, the influence of BMI and our new index in developing postoperative infections is assessed.

## 2. Materials and Methods

### 2.1. Inclusion Criteria

The inclusion criteria for this study were the undergoing of hip prosthesis-related surgery performed at our tertiary center in 2018 and 2019. Patients treated for infections were excluded.

In the years of 2018 and 2019, a total of 921 THA procedures were performed at our tertiary center. All patients undergoing primary arthroplasty or a one-stage revision without intraoperative detection of bacteria were included in this retrospective study. One patient was excluded because of invalid BMI calculations after leg amputation. The patients gave their consent for the anonymized use of their data for research purposes in writing.

Overall, 635 patients were included, of whom 26 underwent more than one surgery during that time. In total, we accounted for 659 interventions. The median age at the time of intervention was 69 (16 to 98) years, with a distribution of 368 (58%) women and 267 (42%) men. The surgeries performed consisted of 573 primary arthroplasties (87%) and 86 revisions (13%).

### 2.2. Data Collection

During preparation for the respective surgeries, the statistical data used in this study were obtained: the date of birth and height were given by each patient, their weight was measured, and the standard X-ray of the pelvis, in the supine position and centered on the symphysis, was reviewed. In 520 (79%) cases, the patients presented themselves for at least one of the scheduled follow-up examinations after six weeks or earlier in the case of problems. The consultations were performed in our outpatient clinic and consisted of questioning as well as physical and radiographic examinations.

For each patient, their BMI was calculated using the common equation [19], the preoperative X-rays were reviewed, and our new obesity index was obtained. Surgical approaches were noted. The standard in our center during this time was the direct anterior approach (DAA) for primary surgeries. In the revisions, the existing incision was reopened when possible; if this was not possible or favorable, a lateral or posterior approach was chosen.

On top of full infections, superficial or deep wounds and prolonged healing (i.e., a residual scab after six weeks) were noted as complications. 

### 2.3. X-ray Grading

**Figure 1:**
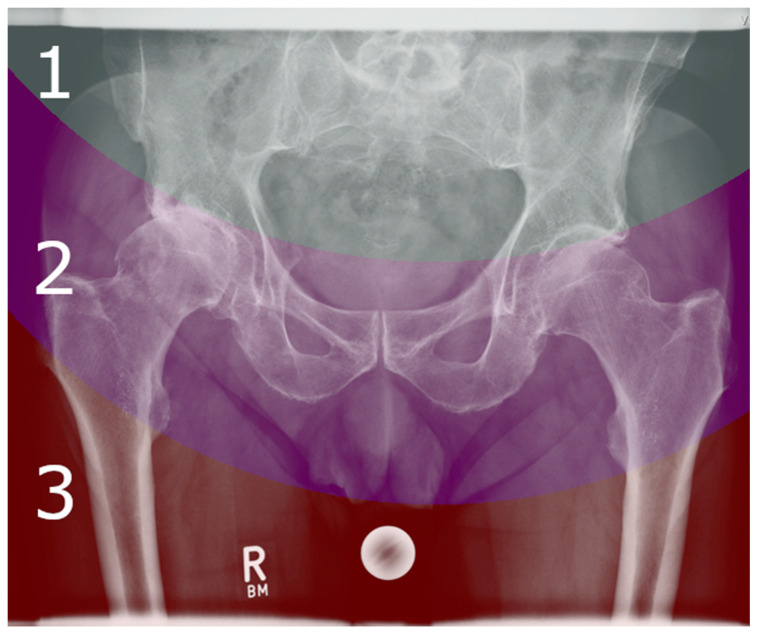
Boundaries when determining our new index: radiograph of the hip, centered on the symphysis. Grading was performed on the left hip with fewer signs of osteoarthritis. If no contour is observable, as in this example, grade 0 is given. A visible contour not projecting on the femur (green) is considered as grade 1. If the fat flap projects on the femur between the head and the lesser trochanter (purple), this results in grade 2. A projection below the lesser trochanter (red) is graded as 3.

Our index is made up of four grades, which can be determined using radiographs of the pelvis, centered on the symphysis (Figure 1), performed on the lying patient. The caudal contour of the belly tissue on these X-rays was observed. 

**Figure 2:**
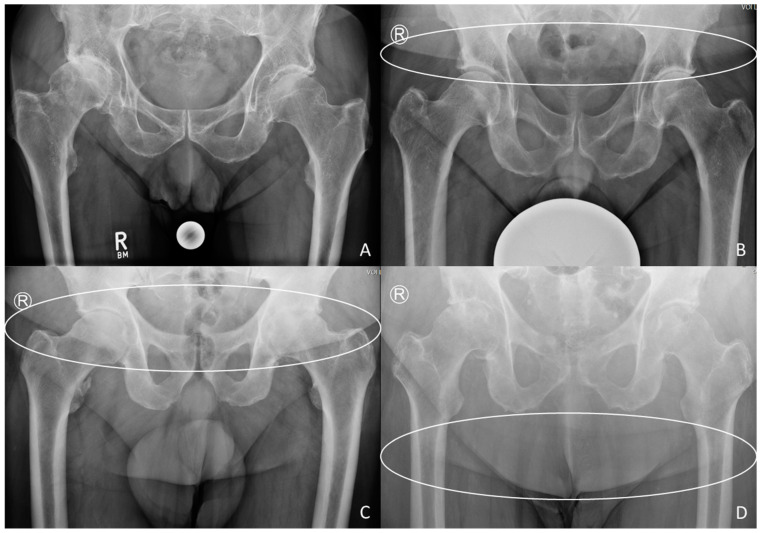
Examples of the grades of our new index: (**A**) grade 0, no contour visible; (**B**) grade 1, contour cranial of the femoral head (white circle); (**C**) grade 2, contour between the femoral head and the lesser trochanter (white circle); (**D**) grade 3, the most caudal contour below the lesser trochanter (white circle).

If no contour is visible, the patient is registered as grade 0 (Figure 2A). An observable contour above the femoral head results in grade 1 (Figure 2B). A projection on the femoral head and neck was defined as grade 2 (Figure 2C), every projection below the lesser trochanter was considered as grade 3 (Figure 2D). A healthy femoral head should be used to determine the margins; when this is not possible (osteoarthritis of both hips or prosthesis in place), the correct thresholds are extrapolated. Caution is advised with very obese patients; when more than one contour is visible, the most caudal is used (Figure 2D).

### 2.4. Statistics

The correlation between BMI and our new index was investigated for all patients by means of spearman correlation, and Chi^2^ tests were used in the followed-up patients to check the parameters against a possible connection with the development of infections. Statistical testing was carried out using SPSS software version 28 (SPSS, Inc., Chicago, IL, USA). *p*-values of less than 0.05 were considered significant.

## 3. Results

Concerning BMI, 33 patients (5%) were underweight, with a value of under 20 kg/m^2^. A normal weight with a BMI between 20 and 25 kg/m^2^ was found in 211 individuals (33%), whereas 389 (62%) were overweight and 163 (26%) were obese at the time of intervention with a BMI of more than 25 and 30 kg/m^2^, respectively. The exact numbers for all cases are shown in Table 1.

When classified by our new index, 455 patients (72%) demonstrated no visible contour in their radiographs (grade 0), 108 (17%) presented as grade 1, and 56 (9%) and 16 (2%) presented as grade 2 and 3, respectively.

Of the 573 primary arthroplasties, 520 (91%) were performed via DAA, and antero-lateral and lateral approaches were used in 24 (4%) and 21 (4%) when a larger incision was necessary (i.e., in fractures or tumor prostheses). A primary posterior approach was used eight times (1%) in very obese patients where the other incisions would lie in an unfavorable position due to fat flaps. 

In only six cases of the anterior approaches (7%) an incision was reopened during revision surgery; the same was carried out in 23 (27%) and 18 (21%) cases for anterolateral and posterior incisions. In 39 patients (45%), a lateral incision was either reopened or created for the sake of better visibility during revision. 

No significant difference in BMI or our new index (*p* = 0.051, *p* = 0.679, respectively) was found between the two types of operations.

Of our patients, 42 reported back with an infection or prolonged healing, representing 6% of all patients or 8% of the followed-up patients. Only two of the patients with revision surgeries reported back with complications. Deep wound infection occurred in 25 cases (4%), and surgical revision was asked for in 31 cases (7%).

**Figure 3:**
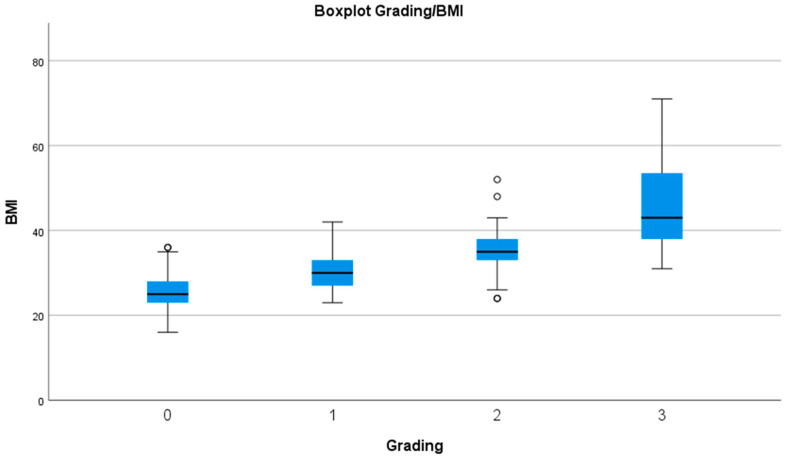
Boxplots of each grade against the BMI.

The correlation between BMI and our new index showed a good rho-value of 0.606 (Spearman) and a *p*-value of <0.0001. The mean BMI of all patients graded as grade 0 was 25.16 (24.83; 25.50) kg/m^2^, for grade 1, it was 30.31 (29.52; 31.09) kg/m^2^, for grade 2, it was 35.06 (33.59; 36.54) kg/m^2^, and it was 45.03 (39.65; 50.41) kg/m^2^ for grade 3, which resulted in significant differences between each grade (Figure 3).

Our new index showed an overall significant difference when compared between the two sexes (*p* = 0.015). In women, a lower mean BMI was shown for each of the grades (Table 2). Grade 0 had a mean BMI of 26.08 (25.63; 26.52) kg/m^2^ in men as opposed to 24.42 (23.96; 24.89) kg/m^2^ in women. Compared grade by grade, only the lowest graduation showed a significant difference between the sexes (Table 2).

No significant differences in any of the other parameters were found between men and women nor between the followed-up patients and the patients with no follow-up.

The patients undergoing primary or revision surgery showed a significant difference in age (*p* = 0.028); the same was found for the position of the incision (*p* = 0.019) and the BMI (*p* = 0.004). No significant connections were found between age and our new index (*p* = 0.633) or the development of postoperative infections (*p* = 0.507).

**Figure 4:**
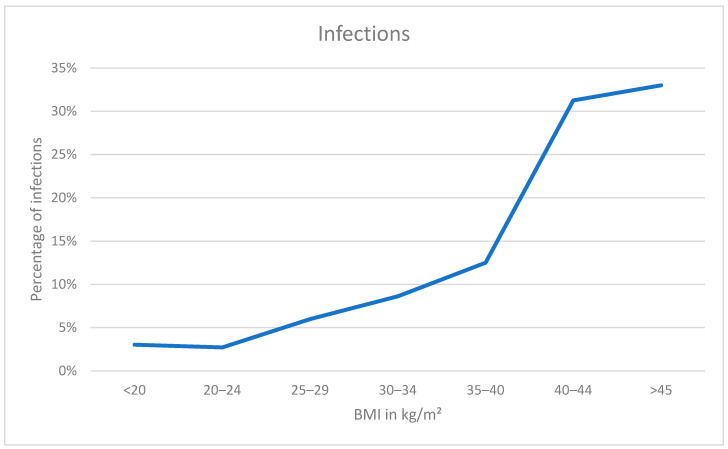
Graph showing infection rates against BMI.

**Figure 5:**
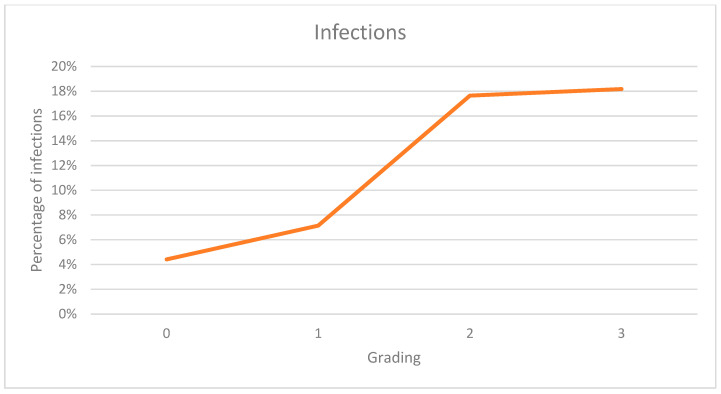
Graph showing infection rates against our index.

The rates of infections in patients against their BMI and our new index are shown in Figure 4 and Figure 5. A higher BMI results in a higher risk of infection. Normal and underweight patients had the lowest rates at 3%, rising with higher values starting at 6% for overweight individuals up to 33% in persons with a BMI over 45. The same trend is shown in the data of our index with 4% of infections in persons with normal radiographs whilst rising from 7% in persons graded as 1 up to 18% in each of the highest categories.

This results in a highly significant difference (*p* < 0.0001) in BMI as well as our new index when compared between the non-infected and infected cohorts. No difference in infection was shown between the different approaches (*p* = 0.693).

These findings are highlighted in Table 3.

## 4. Discussion

In this study, we were able to show that our new index, read from X-rays needed for preoperative planning, can be used to estimate BMI. Furthermore, it can be used to estimate the risk of early postoperative infection in total hip arthroplasty.

Compared to the German average, our cohort included a higher percentage of overweight and obese patients. In this study, 62% were overweight (BMI > 25 kg/m^2^) compared to the average of 52% [2]. The ratio of obesity (BMI > 30 kg/m^2^) was 26% opposed to 19% in the German population [2]. The reason behind this might be the higher rate of osteoarthritis in heavier patients [20].

We compared our primary THA patients to two other collectives: one from the USA and one European group from Sweden, which were observed by Paxton et al. in 2019 [21]. In terms of age, our cohort consisted of a higher percentage of very young (<50 years) and very old (>90 years) patients compared to both of the other groups. The remaining patients compared better with the older European cohort than the American group by age [21]. When looking at BMI distribution, we had a higher percentage of normal and overweight persons (BMI < 30) than both other groups and a higher rate of very obese patients (BMI > 35) than the European control group but less than the US cohort [21].

These differences are probably accounted for by our status as a tertiary center, where more extreme patients both in age and weight are seen. This status results in patients with more comorbidities and more difficult cases.

The major cause of hip arthroplasties worldwide is primary osteoarthritis [22]; this concurs with our data, where 531 patients suffered from this disease (86% of all primary THAs). Our cohort consisted of a comparatively lower number of patients with femoral fractures (1%), which are usually not treated by our department. The biggest difference when compared to a standardized cohort is that our group consisted of 21 patients (4%) that have undergone hip arthroplasties because of cancer-related complications (primary bone cancer around the hip or metastases); fifteen of these patients had received a mega prosthesis. Overall, 7 of the 40 infections after primary surgery developed in non-osteoarthritis patients. 

Considering these circumstances, a higher rate of postoperative complications is to be expected. The treatment algorithm in our center consists of a follow-up 6–8 weeks after implantation. During this visit or during physical rehabilitation, the patients are checked for signs of infection. According to our experience, patients skipping follow-up examinations and not admitted by general practitioners can be viewed as infection-free.

A higher BMI resulting in higher rates of infection is generally considered a fact and is backed up by multiple studies [3,6,13,14,15]. That fact can be validated by our data. Whereas DeMik et al. found little more than a doubled risk of infection in THA patients with a BMI higher than 40 kg/m^2^ compared to a normal group [13], Wagner et al. found a quadrupled rate of infection [14]. The risk in our cohort was 15 times higher (2% to 30%). The small number of cases (*n* = 23 with BMI > 40 kg/m^2^) in this study has to be taken into consideration. A newer study confirmed the higher risk of infection for overweight patients; additionally it was shown that an anterior approach in patients with a BMI > 35 kg/m^2^ resulted in more infections than after the use of the posterior approach [15].

Using our new score, we can show that an approximation of BMI can be obtained by looking at preoperative X-rays. Our results approximately show a mean BMI of 25 kg/m^2^ for grade 0, 30 kg/m^2^ for grade 1, 35 kg/m^2^ in patients graded as 2, and 45 kg/m^2^ for grade 3. The slight difference between the sexes can be explained by the different fat distribution patterns [23]. 

Additionally, to be able to estimate BMI, our study shows that with our tool we can predict higher risks of infection. This helps in assessing individual perioperative risks without the need to obtain the personal data of patients, which they could be unwilling to correctly state, and in patients where a BMI is not obtainable (i.e., amputees).

Our study can give no answer as to the etiology of the surge of infections in overweight individuals. Many reasons have been discussed, for example, deranged immune responses [7] or changes in the skin [8]. 

On top of the generally increased rate of infection in overweight patients after surgery, the placement of the surgical incision could have a worsening effect. Especially when looking at hip arthroplasties, overhanging waists have to be taken into consideration. Flaps over anterior approach areas could cause irritations by sustaining a wet environment and creating mechanical friction around the surgical site. These properties and the closeness to the genital area lead to the perfect breeding ground for bacteria around the wound. DeMik et al. showed a higher effect of BMI concerning infection in total hip replacements compared to total knee replacements [13], which could show the significance of overhanging fat. A work from Shah et al. showed a higher rate of periprosthetic joint infection in anterior approaches compared to posterior approaches in patients with a BMI higher than 35 kg/m^2^ [15].

The proposed grading system could be used to choose an appropriate area not lying under the abdominal fat flap. Further studies are needed to see if a posterior approach could decrease the risk of infection in these patients. Data could also be used to implement a cut-off for performing total hip replacements as suggested by DeMik et al. [16].

Although perioperative antimicrobial prophylaxis is still controversial [24], the findings of this study could help to determine if and how long prophylaxis is administered against the background of the higher prevalence of infection in overweight patients.

Regarding limitations, the retrospective design has to be mentioned, and the inter- and intra-observer reliability were not tested. Furthermore, the patient’s height was not measured; this could lead to report bias by the patients. Minimal differences in this parameter should be neglected for the calculation of BMI. Ideally, a prospective multi-center study should be considered to prove our findings. Additionally, different modes to measure obesity could be applied (i.e., body fat or abdominal girth).

To validate our system, further studies are needed. An additional study is planned to assess the intra- and inter-observer reliability of this study. The difference between the female and the male body should be assessed. Furthermore, a study including more posterior approaches would be interesting in order to assess the influence of the incision site in the equation.

## 5. Conclusions

Our new score enables orthopedic surgeons to assess a patient’s BMI and risk of infection instantly while only looking at pre-existing X-rays.

This study shows that we were able to create a semi-quantitative grading tool for the abdominal contour displayed on preoperative X-rays of the pelvic region for THA planning in order to estimate BMI and therefore the infection rate. A higher abdominal contour grade showed higher infection rates at follow-up.

## Figures and Tables

**Table 1:** Results showing the absolute and relative numbers of patients against the obtained parameters.

		Surgeries		Followed-Up Surgeries		Infections	
		659	Percentage	520	Percentage	42	Percentage
Grading	0	475	72%	370	71%	21	50%
	1	112	17%	95	18%	8	19%
	2	55	8%	41	8%	10	24%
	3	17	3%	14	3%	3	7%
Approaches	DAA	526	80%	420	81%	33	79%
	Antero-lateral	47	7%	39	7%	5	12%
	Lateral	60	9%	40	8%	3	7%
	Posterior	26	4%	21	4%	1	2%
BMI	<20	33	5%	25	5%	1	2%
	20–24	221	33%	169	32%	6	14%
	25–29	234	36%	191	37%	14	33%
	30–34	116	18%	93	18%	10	24%
	35–40	32	5%	25	5%	4	10%
	40–44	16	2%	12	2%	5	12%
	45–49	1	0%	0	0%	0	0%
	>50	6	1%	5	1%	2	5%
Sex	M	280	43%	219	42%	16	38%
	F	379	27%	301	28%	26	62%
Surgery	Primary	573	87%	456	88%	40	95%
	Exchange	86	13%	64	12%	2	5%
Infection	No	617	93%	478	92%		
	Yes	42	7%	42	8%		
	- Follow-up surgery	31	5%	31	6%		
	- Superficial	6	1%	6	1%		
	- Prolonged healing	11	2%	11	2%		

**Table 2:** Results showing the absolute and relative numbers of patients against our index for men and women, as well as the mean BMI and the respective 95% confidence interval for each grade in kg/m^2^.

	Grade	Number of Individuals		Mean BMI	95% Confidence	
Male (*n* = 267)	0	204	76%	26.08	25.63	26.52
	1	45	17%	31.18	29.90	32.47
	2	14	5%	37.78	34.20	41.36
	3	4	2%	47.90	34.32	61.48
Female (*n* = 368)	0	251	68%	24.42	23.96	24.89
	1	63	17%	29.68	28.75	30.61
	2	42	12%	33.85	32.38	35.32
	3	12	3%	44.07	39.40	48.75

**Table 3:** Significances (*p*-values) of all correlations. Obtained using Chi^2^ tests for nominally scaled data and ANOVA correlations for all others. Significant correlations are highlighted in bold.

	BMI	Index	Infect	Age	Sex	Approach
Prim vs. Change	0.113	0.943	0.357	**0.028**	0.379	**<0.0001**
Approach	0.739	0.130	0.693	**0.019**	0.142	
Sex	0.715	**0.015**	0.708	0.576		
Age	0.611	0.633	0.507			
Infect	**<0.0001**	**<0.0001**				
Index	**<0.0001**					

## Data Availability

Data available on request due to privacy and ethical restrictions.

## References

[1] Ng M., Fleming T., Robinson M., Thomson B., Graetz N., Margono C., Mullany E.C., Biryukov S., Abbafati C., Abera S.F. (2014). Global, regional, and national prevalence of overweight and obesity in children and adults during 1980–2013: A systematic analysis for the Global Burden of Disease Study 2013. Lancet.

[2] Schienkiewitz A., Kuhnert R., Blume M., Mensink G.B.M. (2022). Overweight and obesity among adults in Germany—Results from GEDA 2019/2020-EHIS. J. Health Monit..

[3] Yuan K., Chen H.L. (2013). Obesity and surgical site infections risk in orthopedics: A meta-analysis. Int. J. Surg..

[4] Urquhart D.M., Hanna F.S., Brennan S.L., Wluka A.E., Leder K., Cameron P.A., Graves S.E., Cicuttini F.M. (2010). Incidence and risk factors for deep surgical site infection after primary total hip arthroplasty: A systematic review. J. Arthroplast..

[5] Boddapati V., Fu M.C., Tetreault M.W., Blevins J.L., Richardson S.S., Su E.P. (2018). Short-term Complications After Revision Hip Arthroplasty for Prosthetic Joint Infection Are Increased Relative to Noninfectious Revisions. J. Arthroplast..

[6] Huttunen R., Syrjänen J. (2013). Obesity and the risk and outcome of infection. Int. J. Obes..

[7] Martí A., Marcos A., Martínez J.A. (2001). Obesity and immune function relationships. Obes. Rev..

[8] Yosipovitch G., DeVore A., Dawn A. (2007). Obesity and the skin: Skin physiology and skin manifestations of obesity. J. Am. Acad. Dermatol..

[9] Learmonth I.D., Young C., Rorabeck C. (2007). The operation of the century: Total hip replacement. Lancet.

[10] Moldovan F., Moldovan L., Bataga T. (2023). A Comprehensive Research on the Prevalence and Evolution Trend of Orthopedic Surgeries in Romania. Healthcare.

[11] Kelmer G., Stone A.H., Turcotte J., King P.J. (2021). Reasons for Revision: Primary Total Hip Arthroplasty Mechanisms of Failure. J. Am. Acad. Orthop. Surg..

[12] Premkumar A., Kolin D.A., Farley K.X., Wilson J.M., McLawhorn A.S., Cross M.B., Sculco P.K. (2021). Projected Economic Burden of Periprosthetic Joint Infection of the Hip and Knee in the United States. J. Arthroplast..

[13] DeMik D.E., Bedard N.A., Dowdle S.B., Elkins J.M., Brown T.S., Gao Y., Callaghan J.J. (2018). Complications and Obesity in Arthroplasty-A Hip is Not a Knee. J. Arthroplast..

[14] Wagner E.R., Kamath A.F., Fruth K.M., Harmsen W.S., Berry D.J. (2016). Effect of Body Mass Index on Complications and Reoperations After Total Hip Arthroplasty. J. Bone Jt. Surg. Am..

[15] Shah N.V., Huddleston H.P., Wolff D.T., Newman J.M., Pivec R., Naziri Q., Shah V.R., Maheshwari A.V. (2022). Does Surgical Approach for Total Hip Arthroplasty Impact Infection Risk in the Obese Patient? A Systematic Review. Orthopedics.

[16] DeMik D.E., Kohler J.G., Carender C.N., Glass N.A., Brown T.S., Bedard N.A. (2022). What Is the Impact of Body Mass Index Cutoffs on Total Hip Arthroplasty Complications?. J. Arthroplast..

[17] Kragelund C., Omland T. (2005). A farewell to body-mass index?. Lancet.

[18] Maukonen M., Männistö S., Tolonen H. (2018). A comparison of measured versus self-reported anthropometrics for assessing obesity in adults: A literature review. Scand. J. Public Health.

[19] Quetelet A. (1932). Recherches sur le poids de l’homme aux different ages. Nouveaux Mémoirs de L’académie Royale des Sciences et Belles-Lettres de Bruxelles.

[20] Reyes C., Leyland K.M., Peat G., Cooper C., Arden N.K., Prieto-Alhambra D. (2016). Association Between Overweight and Obesity and Risk of Clinically Diagnosed Knee, Hip, and Hand Osteoarthritis: A Population-Based Cohort Study. Arthritis Rheumatol..

[21] Paxton E.W., Cafri G., Nemes S., Lorimer M., Kärrholm J., Malchau H., Graves S.E., Namba R.S., Rolfson O. (2019). An international comparison of THA patients, implants, techniques, and survivorship in Sweden, Australia, and the United States. Acta Orthop..

[22] Havelin L.I., Fenstad A.M., Salomonsson R., Mehnert F., Furnes O., Overgaard S., Pedersen A.B., Herberts P., Kärrholm J., Garellick G. (2009). The Nordic Arthroplasty Register Association: A unique collaboration between 3 national hip arthroplasty registries with 280,201 THRs. Acta Orthop..

[23] Kanehisa H., Miyatani M., Azuma K., Kuno S., Fukunaga T. (2004). Influences of age and sex on abdominal muscle and subcutaneous fat thickness. Eur. J. Appl. Physiol..

[24] Zimmerli W., Trampuz A., Ochsner P.E. (2004). Prosthetic-joint infections. N. Engl. J. Med..

